# RNA sequencing reveals lncRNAs that specifically regulate unsaturated fatty acid generation in buffaloes

**DOI:** 10.5194/aab-69-309-2026

**Published:** 2026-05-26

**Authors:** Xinyu Zeng, Hao Tian, Haopeng Wang, Fangfang Zou, Hongfang Mo, Xingrong Lu, Chunyan Yang, Deshun Shi, Jianghua Shang, Jieping Huang

**Affiliations:** 1 Guangxi Key Laboratory of Animal Breeding, Disease Control and Prevention, College of Animal Science and Technology, Guangxi University, Nanning, Guangxi, 530005, China; 2 Guangxi Key Laboratory of Buffalo Genetics, Reproduction and Breeding, Buffalo Research Institute, Nanning, Guangxi, 530001, China

## Abstract

The content of unsaturated fatty acids (UFAs) is significantly associated with the flavor, taste, and nutritional value of beef. Adipose tissue (AT) develops in multiple depots, and the UFA content of AT varies among different depots. To elucidate the regulatory role of long non-coding RNAs (lncRNAs) in UFA synthesis across different AT depots in buffaloes, we conducted RNA sequencing (RNA-seq) on AT samples from six distinct depots. A total of 8926 lncRNAs were identified and 1363 of them were novel. The numbers of lncRNAs identified in different AT depots were similar. Through weighted gene co-expression network analysis (WGCNA), a module including 207 lncRNAs with a high correlation with UFA content was revealed. Functional enrichment analysis showed that these lncRNAs were significantly enriched in fat deposition and fatty acid metabolism. Notably, two lncRNAs (MSTRG.11229 and MSTRG.16994) were further identified. Both lncRNAs exhibited predominant expression in sternum subcutaneous AT (SSAT) and were upregulated during adipogenic differentiation in SSAT-derived adipocytes. What is more, the expressions of the two lncRNAs presented a high correlation to adipogenesis and UFA synthesis genes as well as UFA content. Collectively, this study provides a comprehensive atlas of lncRNA profiles across six AT depots and identifies two lncRNAs with high correlation with UFA content in buffaloes. These findings offer valuable insights and lncRNAs for the regulation of UFA synthesis in buffaloes.

## 1 Introduction

Fatty acids (FAs) are the major components of adipose tissue (AT), which can be divided into saturated fatty acids (SFAs) and unsaturated fatty acids (UFAs). SFAs are solid or semi-solid at room temperature, and excessive intake easily blocks blood vessels and causes atherosclerosis (de Souza et al., 2015; Hooper et al., 2020). In contrast, UFAs are liquid at room temperature and serve as essential nutrients for humans, contributing to maintaining cell membrane function, reducing blood cholesterol levels, and improving cardiovascular health (Petersen et al., 2024; Nie et al., 2022). Given the widely acknowledged health benefits of UFAs, there is a growing interest in regulating the FA profile of animal-derived products to optimize their nutritional value for consumers. However, in many domestic animal species, the fat is characterized by a relatively high SFA content and a low UFA content (Hennessy et al., 2021). Notably, fat plays a pivotal role in determining the flavor, taste, and juiciness of meat in domestic animals. Specially, intramuscular fat (IMF) content is an important indicator for beef grading. Therefore, revealing important genetic factors involved in UFA synthesis will provide important targets for the genetic improvement of meat quality in cattle.

In domestic animals, AT develops in multiple depots. Based on their anatomical locations, AT can be categorized into subcutaneous AT (SAT), visceral AT (VAT), and IMF. Previous studies have indicated that UFA content varies among different AT depots. Generally, the UFA content in SAT is higher than that in VAT (Jiang et al., 2018; Ferjak et al., 2019; Bartoň et al., 2020; Sobczuk-Szul et al., 2021). In buffaloes, sternum subcutaneous AT (SSAT) exhibits the highest UFA content among SAT depots (Yang et al., 2023). Nevertheless, the underlying mechanisms responsible for the differences in UFA content across distinct AT depots remain to be elucidated, and the key regulatory factors as well as molecular mechanisms involved require further investigation.

Long non-coding RNAs (lncRNAs) are a class of non-coding RNA with a length exceeding 200 nucleotides, which can regulate the expression of their target genes. This regulatory function is achieved either by recruiting regulatory complexes through RNA–protein interactions or by acting as local regulators (Joung et al., 2017). Furthermore, lncRNAs exhibit strong tissue specificity (Muret et al., 2017). Accumulating evidence has demonstrated that lncRNAs modulate the expression of adipogenic genes and lipid-metabolism-related pathways, thereby fat deposition and FA metabolism. For example, lncRNA *NDUFC2-AS* promotes adipogenic differentiation by upregulating the expression levels of thyroid hormone responsive protein (*THRSP*) and CCAAT enhancer binding protein alpha (*C/EBP* α) in buffaloes (Huang et al., 2019b). *lnc210* was demonstrated to facilitate lipid accumulation in buffalo intramuscular adipocytes via upregulating the mRNA expression of peroxisome proliferator-activated receptor γ (*PPARG*) and *C/EBP* α (Zhu et al., 2022). Additionally, lncRNA *BIANCR* was demonstrated to promote the differentiation of bovine intramuscular adipocytes through the extracellular signal-regulated kinase 1/2 (ERK1/2) signaling pathway (Ma et al., 2023), while lncRNA *Hnscr* was demonstrated to regulate adipose lipid metabolism by acting as a mediator of the cyclic AMP/protein kinase A signaling pathway (Guo et al., 2023). Thus, exploring functional lncRNAs in specific AT depots is of great significance for regulating depot-specific adipose development.

Previously, we identified significant differences in the UFA content of AT in different depots in buffaloes (Yang et al., 2023). To elucidate the potential regulatory role of lncRNAs in depot-specific UFA metabolism, in the present study, RNA sequencing (RNA-seq) was performed on samples from six AT depots – including back subcutaneous AT (BSAT), SSAT, inguinal AT (IAT), omental AT (OAT), pericardial AT (PCAT), and perirenal AT (PRAT) – to characterize the lncRNA expression profile across these distinct AT depots in buffaloes. Weighted gene co-expression network analysis (WGCNA) and functional enrichment analysis were employed to identify key lncRNAs involved in UFA synthesis in buffaloes. The results suggest potential targets for the regulation of UFA generation and genetic improvement in the meat quality in buffaloes.

## 2 Materials and methods

### 2.1 Sample preparation

The samples for transcriptome sequencing and FA content were obtained from six Murrah buffaloes (approximately 20 months' old). Details on the age, breed, sex, and body weight of the six Murrah buffaloes were presented in our previous study (Yang et al., 2023). The AT samples for SSAT preadipocyte isolation and qRT-PCR analysis were obtained from three additional Murrah buffaloes, which were 24 months of age and weighed approximately 330 kg. Following the electrical stunning and slaughter of the three buffaloes, ATs from three subcutaneous depots (BSAT, SSAT, and IAT) and three visceral depots (OAT, PCAT, and PRAT) were immediately collected. The BSAT was sampled between the 12th and 13th ribs. For preadipocytes isolation, fresh SSAT was transported to the laboratory in sterile centrifuge tubes containing phosphate-buffered saline (PBS). For qRT-PCR assay, AT samples were immediately placed in liquid nitrogen, transported to the laboratory, and subsequently stored at - 80 °C. All animals were raised in accordance with the standardized management plan of the Buffalo Research Institute of the Chinese Academy of Agricultural Sciences (Nanning, Guangxi, China).

### 2.2 RNA-seq data sources

The RNA-seq data were deposited in the Genome Expression Omnibus (GEO) database of the National Center for Biotechnology Information (NCBI) (https://www.ncbi.nlm.nih.gov/geo/, last access: 29 April 2026) under the accession number GSE208541 (Yang et al., 2023). AT samples from six depots were collected from each of the six buffaloes (36 AT samples in total). Total RNA was extracted, and strand-specific rRNA-depleted libraries were constructed for each sample and sequenced on the Illumina HiSeq 4000 platform (Illumina, San Diego, CA, USA)

### 2.3 Quality control and lncRNA prediction

The sequencing primers, connectors, and low-quality reads were removed from the raw data using the fastp software (Yang et al., 2023). The fastp-filtered data were aligned to the rRNA database using Bowtie 2 software (Langmead and Salzberg, 2012) to eliminate rRNA-derived reads. The resulting clean reads were mapped to the buffalo reference genome (GCF_019923935.1) using HISAT2 (Kim et al., 2015), and the mapped reads were assembled and quantified by StringTie (Pertea et al., 2015), which calculates fragments per kilobase of transcript per million fragments mapped (FPKM) to assess transcript expression levels (Trapnell et al., 2010). After excluding known mRNAs and transcripts shorter than 200 bp, the remaining transcripts were analyzed using four prediction tools: CPC2 (Kang et al., 2017), CNCI (Sun et al., 2013), PFAM (Mistry et al., 2020), and EGGNOG (Cantalapiedra et al., 2021). Transcripts with ≥ 2 exons and a length > 200 nt were defined as lncRNAs. The gffcompare software (Pertea and Pertea, 2020) was used to compare with known transcripts.

### 2.4 Weighted gene co-expression network analysis

WGCNA was performed using the R package (Li et al., 2020c). Based on the previously identified lncRNAs, those with FPKM > 0 (4655 transcripts) were selected as input data. WGCNA was performed in conjunction with ATs from different depots and their components (triglyceride, TG; SFAs; UFAs). A soft threshold ( β ) of 3 was selected based on the scale-free network principle and one-step method, with a minModuleSize of 50 and mergeCutHeight of 0.5. By analyzing the correlation between phenotypes and modularized lncRNAs, relevant co-expression modules and candidate lncRNAs were identified.

### 2.5 lncRNA target gene prediction and functional enrichment

Cis-acting target genes of lncRNAs were predicted by searching within 10 kb upstream and downstream of each lncRNA using NCBI resources. Subsequently, transacting target genes were predicted by analyzing the correlation between the expression levels of candidate lncRNAs and mRNAs using R software. Pearson's correlation coefficient ( r ) was used to evaluate the correlation between lncRNA and mRNA expression levels, with statistical significance set at p < 0.05. The R package was used to perform Gene Ontology (GO) (human, http://www.geneontology.org/, last access: 29 April 2026) and Kyoto Encyclopedia of Genes and Genomes (KEGG) (human, https://www.kegg.jp/, last access: 29 April 2026) enrichment analysis of the lncRNA target genes. A p < 0.05 indicated a significant relationship between the terms and the lncRNA target genes.

### 2.6 Preadipocyte isolation and culture

The preadipocytes were isolated from buffalo SSAT using the tissue block method as previously described (Zhu et al., 2023a). The preadipocytes were cultured in a humidified incubator at 37 °C and 5 % CO_2_ with high-glucose Dulbecco's Modified Eagle Medium (DMEM; HyClone, Logan, UT, USA), supplemented with 10 % fetal bovine serum (FBS) and 1 % penicillin-streptomycin (Gibco, Grand Island, NY, USA).

### 2.7 Adipogenic differentiation and oil red O staining

The preadipocytes were seeded at a density of 40 % and induced to undergo adipogenic differentiation when reaching 90 % confluence, following the methodology previously described (Huang et al., 2020). The cells were first cultured in ab induction medium for 2 d, after which the medium was replaced with maintenance medium until visible lipid droplets appeared. To evaluate the differentiation efficiency, oil red O staining was performed as previously outlined (Huang et al., 2019b). Briefly, the adipocytes were washed three times with PBS and fixed with 10 % formalin for 1 h. After fixation, cells were rinsed with 60 % isopropyl alcohol and stained with 0.3 % oil red O solution for 20 min. Finally, the cells were washed thoroughly with PBS and observed under a light microscope.

### 2.8 Total RNA extraction and reverse transcription

Total RNA was extracted from the samples using TRIzol reagent (GenStar, Beijing, China), according to the manufacturer's instructions. Complementary DNA (cDNA) was synthesized by reverse transcription using StarScript III All-in-one RT Mix with gDNA Remover (GenStar, Beijing, China).

### 2.9 qRT-PCR assay

qRT-PCR assay was performed using SYBR Premix (GenStar, Beijing, China) on a Roche LightCycler 96 Real-Time PCR System. The cycling conditions were as follows: 37 °C for 2 min, 95 °C for 2 min, followed by 45 cycles of 95 °C for 10 s and 60 °C for 30 s. All reactions were performed in triplicate. β *-actin* was used as the internal reference gene, and specific primers were designed using Primer Premier 5.0 software (Supplement Table S1). The relative expression levels of the candidate genes were calculated using the cyclic threshold method (2 ${}^{-\Delta \Delta \mathrm{Ct}})$ .

### 2.10 Statistical analysis

One-way analysis of variance (ANOVA) was used for statistical comparisons using SPSS software version 26.0 (IBM, Armonk, NY, USA). A value of p < 0.05 was considered statistically significant. Linear regression analysis was conducted to evaluate the correlation between UFA content and gene expression levels, with the coefficient of determination ( $R^{2}$ ) and Pearson correlation coefficient ( r ) used to identify significant correlations in accordance with the aforementioned statistical threshold. Data visualization and additional analyses were conducted using GraphPad Prism 8.0.

## 3 Results

### 3.1 Identification of lncRNAs in six AT depots

**Figure 1:**
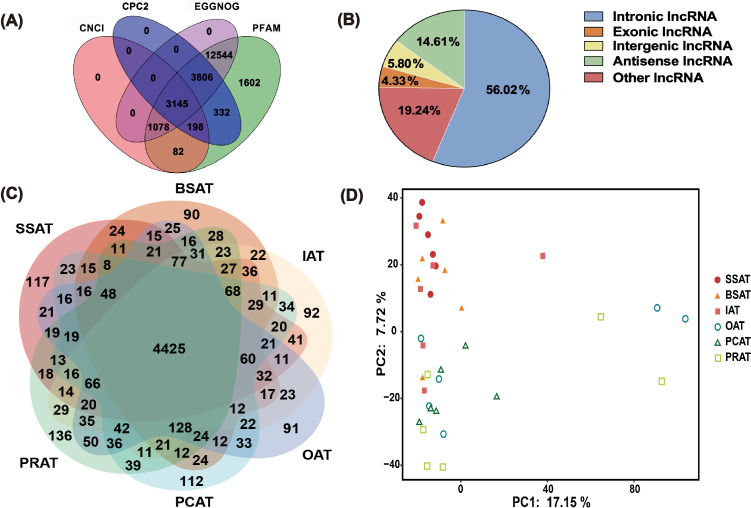
lncRNA expression analysis in six different AT depots in buffaloes based on RNA-Seq data. **(A)** Venn diagram showing the overlap of novel lncRNA transcripts predicted by four tools (CPC2, CNCI, PFAM, EGGNOG). **(B)** Classification and proportional distribution of 1363 novel lncRNAs. **(C)** Venn diagram illustrating the distribution of total lncRNAs (8926) across six AT depots. **(D)** PCA scatter plot of the 36 AT samples. Principal component 1 (PC1, x axis) explains 17.15 % of the variance, and principal component 2 (PC2, y axis) explains 7.72 % of the variance. SSAT, sternum subcutaneous adipose tissue; BSAT, back subcutaneous adipose tissue; IAT, inguinal adipose tissue; OAT, omental adipose tissue; PCAT, pericardial adipose tissue; PRAT, perirenal adipose tissue.

The transcriptome data from three SAT depots and three VAT depots of six buffaloes were analyzed. A total of 5.3 billion 100 nt paired-end clean reads were obtained (Table S2). More than 94 % of the reads were mapped to the buffalo reference genome (Table S2). After excluding known mRNAs and transcripts smaller than 200 bp, four prediction tools, including CPC2 (Kang et al., 2017), CNCI (Sun et al., 2013), PFAM (Mistry et al., 2020), and EGGNOG (Cantalapiedra et al., 2021), were used to filter out transcripts with coding potential. As a result, a total of 8926 transcripts were identified as lncRNAs (Table S3). Among these, 3145 transcripts (corresponding to 1363 lncRNAs) were consistently predicted as novel lncRNA transcripts (Fig. 1A, Table S4). Among the novel lncRNAs, intronic lncRNA were the most abundant, accounting for 56.02 % of the total; this was followed by other lncRNA (19.24 %), antisense lncRNA (14.6 %), intergenic lncRNA (5.8 %), and exonic lncRNA (4.3 %, the least abundant; Fig. 1B, Table S5). Ultimately, 1363 novel lncRNAs were identified (Table S5).

Of the 8926 total lncRNAs, 4425 were detected in all of the six AT depots. There, 117, 90, 92, 91, 112, and 136 lncRNAs were specifically expressed in SSAT, BSAT, IAT, OAT, PCAT, and PRAT, respectively (Fig. 1C, Table S6). These results indicated significant difference in lncRNA expression profiles among AT depots, which may be attributed to the diversity in components between AT depots. Principal component analysis (PCA) of all identified lncRNAs generally separated SATs from VATs (Fig. 1D), suggesting depot-specific lncRNA expression patterns (Fig. 1D).

### 3.2 Weighted gene co-expression network analysis

**Figure 2:**
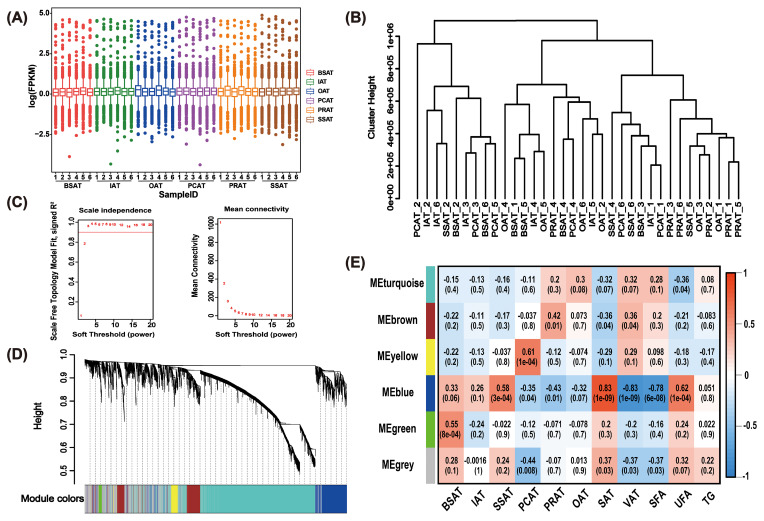
WGCNA analysis reveals co-expression modules highly associated with UFAs. **(A)** Box plot illustrates the expression levels of lncRNA in 36 AT samples through FPKM values. **(B)** Cluster analysis using transcripts with average FPK > 0 across the 34 AT samples. Two samples (SSAT-1 and SSAT-3) deviating from others were removed. **(C)** Network topology for various soft-thresholding powers. **(D)** Gene dendrogram clustering the dissimilarity, based on the consensus topological overlap. Modules are indicated by the color row. **(E)** Relationships between modules and traits. Each cell presents the correlation coefficient between module (row), trait (column), and p value (in parentheses). Modules are indicated by the color column, and the number in the corresponding color indicates the number of genes involved in the corresponding module. SSAT, sternum subcutaneous adipose tissue; BSAT, back subcutaneous adipose tissue; IAT, inguinal adipose tissue; OAT, omental adipose tissue; PCAT, pericardial adipose tissue; PRAT, perirenal adipose tissue; SAT, subcutaneous adipose tissue; VAT, visceral adipose tissue; SFA, saturated fatty acids; UFA, unsaturated fatty acids; TG, triglyceride.

WGCNA was performed to investigate the correlation between lncRNA expression and AT depots, TG content, SFA content, and UFA content (Fig. 2). Notably, two outlier samples (SSAT-1 and SSAT-3) were excluded based on sample clustering analysis, and 34 AT samples were ultimately used for WGCNA (Fig. 2A–B). Based on the scale-free network topology criterion, the soft threshold ( β ) was set to 3 (Fig. 2C). The 8926 lncRNAs were assigned to six co-expression modules based on their expression patterns across the six AT depots (Fig. 2D). Subsequently, correlation analysis was conducted between 11 phenotypic traits of buffaloes and the six co-expression modules (Fig. 2E). As a result, only the MEblue module exhibited significant positive correlation with UFA content ( r= 0.62, p= 1e - 04). Notably, the MEblue module was also strongly positively correlated with SAT ( r= 0.83, p= 1e - 09) and SSAT ( r= 0.58, p= 3e - 04). In addition, the MEblue module presented a significant negative correlation with SFA content ( r= - 0.78, p= 6e - 08) and a strong negative correlation with VAT ( r= - 0.83, p= 1e - 09) (Fig. 2E).

### 3.3 Functional enrichment analysis of the lncRNAs in the MEblue module

To identify lncRNAs that have potential regulatory roles in UFA synthesis, we further investigated the 596 lncRNAs in the MEblue module (Table S7). A correlation analysis was first conducted between the expression levels of lncRNAs in the MEblue module and UFA content across the six AT depots. As a result, a total of 207 lncRNAs with r > 0.85 were identified (Table S8). Then, 1181 target genes of the 207 lncRNAs were predicted using R software (Table S9). These target genes include many genes involved in adipogenesis and UFA synthesis, such as integrin-beta1 (*ITGB1*), prostaglandin E synthase (*PTGES*), ectonucleotide pyrophosphatase/phosphodiesterase 2 (*ENPP2*), cluster of differentiation 109 (*CD109*), mitochondrial tryptophanyl-tRNA synthetase (*WARS2*), *T-box 15* (*TBX15*), and *C1q/TNF*-related protein 1 (*C1QTNF1*). Certainly, numerous other functional genes were also targeted, such as *PDGFA* and *TGFBR2*, which are associated with the regulation of mesenchymal cell proliferation; and *NUMB*, which is involved in the regulation of nervous system development (Tables S9 and S10).

**Figure 3:**
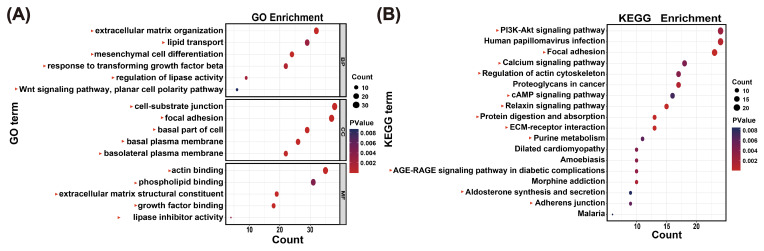
Functional enrichment analysis reveals the functions of lncRNA. **(A)** GO enrichment analysis by lncRNAs in the MEblue module, with a correlation greater than 0.85 to UFAs. Red triangles indicate those items associated with UFA synthesis. **(B)** KEGG enrichment analysis by lncRNAs in the MEblue module, with a correlation greater than 0.85 to UFAs. Red triangles indicate those items associated with UFA synthesis.

To systematically explore the potential functions of the 207 lncRNAs, the 1181 target genes were used for functional enrichment analysis. GO analysis indicated that target genes of the 207 lncRNAs were associated with lipid transport, lipid droplets, and ECM (Table S10; Fig. 3A). KEGG analysis identified adipogenesis-related pathways, such as the PI3K-Akt signaling pathway (Table S11; Fig. 3B). These results indicate that lncRNAs in the MEblue module are associated with adipogenesis in buffaloes. In addition, pathways associated with the growth and development of muscle tissue were also enriched, including the regulation of actin cytoskeleton and cytoskeleton in muscle cells. Certainly, various other functional pathways have also been enriched (Table S11).

### 3.4 Screening of key lncRNAs associated with UFA synthesis

**Figure 4:**
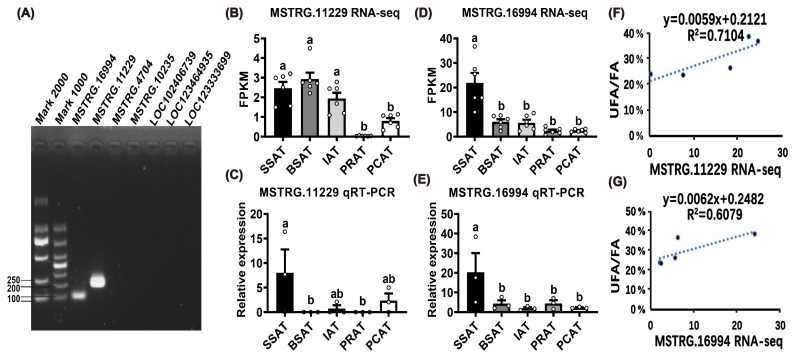
Identification of RNA-seq results for MSTRG.11229 and MSTRG.16994. **(A)** Validation of qRT-PCR primers of the candidate lncRNAs. **(B)** and **(D)** expression profiles of MSTRG.11229 and MSTRG.16994 across five AT depots based on RNA-seq data. **(C)** and **(E)** expression profiles of MSTRG.11229 and MSTRG.16994 across five AT depots based on qRT-PCR detection. The relative expression of lncRNAs was normalized to β *-actin,* with SSAT as the control group. **(F)** and **(G)** correlation analysis between MSTRG.11229 and MSTRG.16994 expression level (RNA sequencing), and UFA content across the five AT depots. Data are presented with mean ± SEM. The lowercase letters indicate significant difference, p < 0.05.

To further identify functional lncRNAs in the MEblue module that are associated with UFA synthesis, seven lncRNAs with FPKM > 2 among the 207 UFA-correlated lncRNAs were randomly selected for expression validation by qRT-PCR (Table S12). However, only two lncRNAs were successfully validated for specific quantitative primers, namely MSTRG.11229 and MSTRG.16994 (Fig. 4A). Five other lncRNAs failed to be detected, which may be due to their low endogenous expression levels. Based on RNA-seq data, MSTRG.11229 had a higher expression in SATs than that in VATs (Fig. 4B). qRT-PCR results confirmed that MSTRG.11229 exhibited the highest expression level in SSAT (Fig. 4C). Similarly, both RNA-seq and qRT-PCR data demonstrated that MSTRG.16994 has the highest expressed in SSAT (Fig. 4D, E). Notably, both MSTRG.11229 and MSTRG.16994 showed a high correlation with UFA content across the six AT depots (Fig. 4F, G). Collectively, these findings suggest that MSTRG.11229 and MSTRG.16994 may be key regulators involved in the regulation of UFA synthesis in buffaloes.

**Figure 5:**
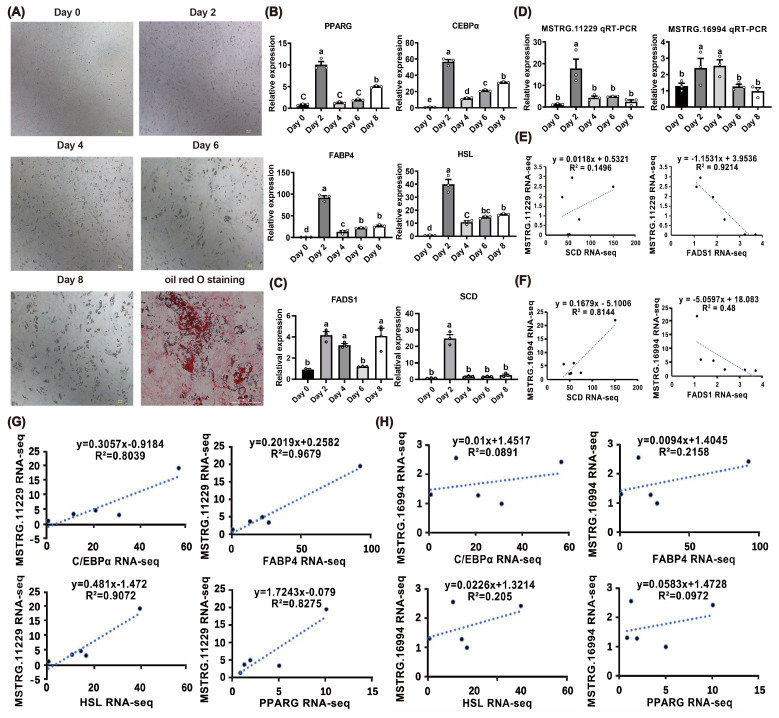
Expression profiles of MSTRG.11229 and MSTRG.16994 and their correlation with UFA synthesis. **(A)** Micrograph of buffalo SSAT preadipocytes during adipogenic differentiation (days 0–8). Oil red O staining was performed on day 8. Scale bar = 100 µ m. **(B–D)** Expression levels of adipogenic marker genes (*PPARG*, * C/EBP* α , *FABP4*, *HSL*), UFA synthesis marker genes (*SCD, FADS1*), MSTRG.11229, and MSTRG.16994 during differentiation. The relative expression of adipogenic marker genes was normalized to β *-actin,* with day 0 as the control group. **(E)** Correlation analysis of expression levels between MSTRG.11229 and *SCD/FADS1* based on RNA-seq data. **(F)** Correlation analysis of expression levels between MSTRG.16994 and *SCD/FADS1* based on RNA-seq data. **(G)** Correlation analysis of expression levels between MSTRG.11229 and adipogenic marker genes (*PPARG*, *C/EBP* α , *FABP4*, *HSL*) based on RNA-seq data. **(H)** Correlation analysis of expression levels between MSTRG.16994 and adipogenic marker genes (*PPARG*, *C/EBP* α , *FABP4*, *HSL*) based on RNA-seq data. Data are presented with mean ± SEM. The lowercase letters indicate significant difference, p < 0.05; the uppercase letters indicate a highly significant difference, p < 0.01.

To further investigate the significant roles of MSTRG.11229 and MSTRG.16994 in UFA synthesis, preadipocytes were isolated from buffalo SSAT, which is a tissue type significantly associated with UFA content. SSAT-derived preadipocytes were induced to undergo adipogenic differentiation. As shown in Fig. 5A, lipid droplets gradually accumulated during the process of adipogenic differentiation. The typical adipogenic markers *PPARG* (Zhu et al., 2023b), *C/EBP* α (Tian et al., 2022), fatty acid binding protein 4 (*FABP4*) (Rodríguez-Calvo et al., 2019), and hormone-sensitive lipase (*HSL*) (Fang et al., 2017) exhibited significant upregulation on day 2 of the adipogenic differentiation (Fig. 5B). These results indicated that SSAT-derived preadipocytes were well induced to adipogenic differentiation. In addition, the expression levels of stearoyl-CoA desaturase (*SCD*) and fatty acid desaturases 1 (*FADS1*), marker genes of UFA synthesis, were upregulated on day 2 of the adipogenic differentiation (Fig. 5C). Consistently, MSTRG.11229 and MSTRG.16994 were upregulated on day 2 of the adipogenic differentiation (Fig. 5D). Notably, MSTRG.11229 exhibited high correlations with *FADS1*, and MSTRG.16994 exhibited high correlations with *SCD* (Fig. 5E, F). MSTRG.11229 was strongly correlated with adipogenic markers (*PPARG*, *C/EBP* α , *FABP4*, *HSL*), whereas no such strong correlation was observed for MSTRG.16994. Interestingly, both of them displayed the highest correlation with *FABP4* of adipogenic markers (Fig. 5G–H). Collectively, these findings further indicate that MSTRG.11229 and MSTRG.16994 are significant regulators involved in UFA synthesis in buffaloes.

## 4 Discussion

UFA content plays a significant role in the meat quality of livestock animals. Revealing key lncRNAs involved in UFA synthesis can provide valuable targets for the genetic improvement of meat quality in livestock. UFA content varies across different AT depots in livestock, but the underlying regulatory mechanisms remain largely unclear. In this study, (1) we characterized the lncRNA expression profiles across six AT depots in buffaloes and found that the number of lncRNA expressed in the six AT depots was nearly the same, (2) WGCNA revealed a group of lncRNAs that exhibited high correlation with UFA content, and (3) MSTRG.11229 and MSTRG.16994 are promising regulators involved in UFA synthesis in buffaloes.

There are significant differences in lipid accumulation and metabolism among ATs in different depots, which leads to differences in UFA content in ATs from different depots. Phenotypic differences are essentially driven by differences in gene expression. With the advancement of high-throughput sequencing technology, expression profiles of coding and non-coding transcripts in ATs from different depots have been characterized in pigs (Jin et al., 2021), sheep (Ahmad et al., 2023), cattle (Fiallo Diez et al., 2024; Tan et al., 2025), and buffaloes (Huang et al., 2019a; Huang et al., 2019b; Huang et al., 2019c; Huang et al., 2020). Notably, most of the existing studies mainly focus on two depots or two kinds of AT, such as SAT and VAT. In fact, characterizing AT into SAT and VAT is not sufficient (Yang et al., 2023; Jin et al., 2021). In the present study, we characterized lncRNA expression profiles of ATs from six different depots. A total of 8926 lncRNAs were identified and 1363 of them were novel lncRNAs (Table S3). Previously, 9494 lncRNAs were identified in BSAT of Xinyang buffaloes (swamp-type buffaloes) (Huang et al., 2019b). This difference in quantity may be due to the different reference genome used in the present study (buffalo genome) and the previous study (cattle genome). The numbers of lncRNAs identified in different AT depots were similar, which is consistent with findings in a previous study on mRNA in buffaloes (Yang et al., 2023). The lncRNA expression profiles in the present study have enhanced our understanding of the transcriptional expression characteristics of ATs from different depots of river buffalo.

WGCNA is a biological network analysis method that mitigates multiple testing issues in large-scale data analysis by leveraging global gene expression patterns (Pan et al., 2022). In a co-expression network, WGCNA identifies modules, explores the relationship between modules and external information (such as traits, pathways, and SNPs), and measures the relationship between genes and modules. It has been widely used to reveal genes associated with complex traits in humans (Xu et al., 2023), model animals (Liu et al., 2021), and livestock animals (Yu et al., 2025; Zhang et al., 2023). For example, WGCNA identified *ITGB1* as a critical gene associated with the IMF content in beef cattle (Yu et al., 2025). Similarly, multiple important mRNAs, circRNAs, and lncRNAs were found to be related to intramuscular adipogenesis in cattle (Yang et al., 2022). In buffaloes, several key genes (Wang et al., 2022) and circMARK3 (Feng et al., 2022) were identified to be associated with fat deposition in buffaloes via WGCNA. In the present study, WGCNA identified the MEblue module, whose lncRNAs exhibit a strong positive correlation with UFA content ( r= 0.62, p= 1e - 04) (Fig. 2E). To the best of our knowledge, this is the first set of lncRNAs associated with UFA synthesis in buffaloes. Additionally, the MEblue module is strongly positively correlated with SAT ( r= 0.83, p= 1e - 09) and SSAT ( r= 0.58, p= 3e - 04), and strongly negatively correlated with VAT ( r=- 0.83, p= 1e - 09) and SFA content ( r= - 0.78, p= 6e - 08) (Fig. 2E). Consistent with previous studies, SAT depots generally have higher UFA content than that in VAT depots (Ferjak et al., 2019; Bartoň et al., 2020; Sobczuk-Szul et al., 2021), and SSAT has the highest UFA content among buffalo SAT depots (Yang et al., 2023). These results further support a strong positive correlation between UFA content and SSAT, suggesting that MEblue module lncRNAs may play crucial roles in buffalo UFA synthesis.

Existing research has shown that lncRNAs modulate gene expression at multiple regulatory layers (Ponjavic et al., 2007). Functionally, lncRNAs are classified as cis-acting or transacting. Cis-acting lncRNAs regulate the transcription of neighboring genes by recruiting or displacing transcription factors and chromatin remodeling enzymes at regulatory regions, thereby affecting the activation or repression of adjacent gene promoters (Engreitz et al., 2016). In contrast to cis-acting lncRNAs, transacting lncRNAs dissociate from the primary locus of transcription and regulate gene expression from a great distance (Herman et al., 2022). To explore the potential functions of the 207 lncRNAs in the MEblue module, we predicted both cis- and transacting target genes, including UFA synthesis-related genes such as *PTGES* and *ENPP2* (Table  S9). *PTGES* is a key enzyme for prostaglandin E_2_ (PGE_2_) synthesis in the arachidonic acid pathway (Wang et al., 2019). *ENPP2* activates the SREBP-1/FAS signaling pathway to promote FA synthesis (Lu et al., 2024). In addition, functional enrichment indicated that the 207 lncRNAs were significantly associated with FA metabolism-related pathways, including the PI3K-Akt signaling pathway, calcium signaling pathway, and cyclic adenosine monophosphate (cAMP) signaling pathway (Fig. 3; Table S11). Previous studies have shown that the PI3K-Akt signaling pathway is closely related to adipogenesis and UFA synthesis (Li et al., 2020b). Inhibiting the activity of PI3K and Akt will block the proliferation of preadipocytes (Zhang et al., 2021). Besides, the PI3K-Akt pathway mediates UFA synthesis by regulating *SREBP* activity (Fontana et al., 2024; Liu et al., 2025). In addition, the PI3K-Akt pathway can also mediate de novo lipid synthesis by regulating *SREBPs*, a positive regulator for UFA synthesis (Fontana et al., 2024; Liu et al., 2025). Sustained cytosolic Ca^2+^ elevations potentiate cAMP production, which activates specific lipases to regulate FAs metabolism via the CREB-PPAR α -PGC1 α transcriptional pathway (Filadi et al., 2023). While numerous other functional genes and pathways were also targeted and enriched, the aim of the present study is to reveal lncRNAs involved in UFA synthesis. Thus, we mainly focused on the genes and pathway associated with UFA synthesis.

To further reveal the functional lncRNAs involved in UFA synthesis, seven lncRNAs with FPKM > 2 among the 207 lncRNAs were randomly selected for qRT-PCR validation (Fig. 4A; Table S1). Among them, five lncRNAs failed to be identified, which may due to their low expression levels in true or the relatively low quality of the buffalo genome used. Meanwhile, many lncRNAs are either undetectable or sporadically expressed (Groeneweg et al., 2020). Two other lncRNAs – MSTRG.16994 and MSTRG.11229 – were successfully identified by qRT-PCR (Fig. 4A). Both of them were upregulated in the early stages of adipogenic differentiation, indicating their involvement in adipogenesis. The predicted target gene of MSTRG.16994, *CD109* (Table S9), is a glycosylphosphatidylinositol (GPI)-anchored protein and serves as a co-receptor for transforming growth factor- β (TGF- β ), which plays a pivotal role in regulating the differentiation and lipid accumulation of MSCs (Cristancho and Lazar, 2011; Sun et al., 2024). Therefore, MSTRG.16994 may regulate fat deposition by modulating the TGF- β signaling pathway. Similarly, two predicted target genes of MSTRG.11229 – *WARS2* and *TBX15* (Table S9) – are associated with adipogenic differentiation and lipid metabolism. *TBX15* is a significant transcriptional regulator reversing the expression of pivotal adipogenic genes as *PPARG*, peroxisome proliferator-activated receptor alpha (*PPARA*), and adiponectin (*ADIPOQ*) (Pan et al., 2021). *WARS2* has been identified as a potential regulator of fat distribution (Mušo et al., 2022). The trans-target gene of MSTRG.11229, *C1QTNF1*, is an adipokine that prevents body weight gain induced by high-fat feeding in mice (Ren et al., 2022). Therefore, MSTRG.11229 may regulate lipid metabolism by modulating the expression of pivotal adipogenic genes and the secretion of adipokines.

Both MSTRG.11229 and MSTRG.16994 presented a high correlation with adipogenesis and UFA synthesis genes. In particular, both of them exhibited the highest correlation with lipid chaperone *FABP4* of four adipogenic markers (Fig. 5G, H). *FABP4* is a typical intracellular lipid chaperone, primarily secreted by adipocytes and macrophages. It is involved in promoting the processes of lipid storage, distribution, transport, breakdown, and metabolism (Li et al., 2020a). Through interactions with *PPARG* and *HSL*, *FABP4* maintains adipocyte homeostasis and regulates adipocyte metabolism (Prentice et al., 2019). Recent studies in buffalo mammary epithelial cells (BMECs) have shown that the overexpression of two *PPARG* isoforms (*PPAR* γ *-X17* and *PPARG-X21*) can significantly reduce the levels of SFAs while increasing the content of UFA, particularly the monounsaturated fatty acids (MUFAs) content (Wang et al., 2024). Meanwhile, studies have shown that promoting the expression of *PPARG* can enhance UFAs and triacylglycerol (TAG) synthesis (Li et al., 2023). *HSL* is an intracellular lipase that is responsible for catalyzing the hydrolysis of various substrates, including TAG, diacylglycerol (DAG), and monoacylglycerol (MAG) (Althaher, 2022). An increasing body of evidence suggests that the role of *HSL* as a DAG hydrolase is more significant (Lampidonis et al., 2011). Studies have shown that silencing Msi2 can increase the proportion of UFAs (Lyu et al., 2023). Moreover, studies have found that the physicochemical properties of DAG exhibit a high content of UFA, particularly oleic acid and linoleic acid. Therefore, MSTRG.11229 and MSTRG.16994 may have a significant impact on the regulation of UFA generation.

## 5 Conclusions

This study provides a comprehensive atlas of lncRNA profiles across six AT depots in buffaloes. WGCNA revealed 207 lncRNAs highly correlated with UFA content. Notably, MSTRG.11229 and MSTRG.16994 exhibited predominant expression in SSAT and were upregulated during adipogenic differentiation, showing strong correlation with UFA content ( r > 0.85). These findings not only enhance our understanding of the regulatory roles of lncRNAs in fat deposition and UFA synthesis but also provide valuable candidate lncRNAs for future studies aiming to improve meat quality in buffaloes.

## Supplement

10.5194/aab-69-309-2026-supplement The supplement related to this article is available online at https://doi.org/10.5194/aab-69-309-2026-supplement.

## Data Availability

The RNA sequencing data used in this study are publicly deposited in NCBI's Genome Expression Omnibus (GEO) (https://www.ncbi.nlm.nih.gov/geo/, last access: 29 April 2026). The accession number is GSE208541. Upon reasonable request, the corresponding authors will make available any underlying data relevant to the article.
