# Supplementary material for: RNA sequencing reveals lncRNAs that specifically regulate unsaturated fatty acid generation in buffaloes
Source: Arch Anim Breed. 2026 May 26;69(2):309–21. doi: 10.5194/aab-69-309-2026 (PMC13224979; doi:10.5194/aab-69-309-2026)
Supplement: The supplement related to this article is available online at https://doi.org/10.5194/aab-69-309-2026-supplement. [file aab-69-309-2026-supplement.zip › aab-69-309-2026-supplement-title-page.pdf]

Supplement of Arch. Anim. Breed., 69, 309–321, 2026
https://doi.org/10.5194/aab-69-309-2026-supplement
© Author(s) 2026. CC BY 4.0 License.

Open Access
Archives Animal Breeding

Supplement of

RNA sequencing reveals lncRNAs that specifically regulate unsaturated fatty acid generation in buffaloes

Xinyu Zeng et al.

Correspondence to: Jieping Huang (huangjieping@gxu.edu.cn)

- aab-69-309-2026-supplement-title-page.pdf
- Table S1.xlsx
- Table S10.xlsx
- Table S11.xlsx
- Table S12.xlsx
- Table S2.xlsx
- Table S3.xlsx
- Table S4.xlsx
- Table S5.xlsx
- Table S6.xlsx
- Table S7.xlsx
- Table S8.xlsx
- Table S9.xlsx

The copyright of individual parts of the supplement might differ from the article licence.
